# Safety and effect of intermittent intrapulmonary percussive ventilation on oxygen saturation and hemodynamic functions

**DOI:** 10.1186/cc10729

**Published:** 2012-03-20

**Authors:** I Blum, R Janssen-Dean, A Van Overdijk, B Speelberg

**Affiliations:** 1 St Anna Hospital Geldrop, the Netherlands

## Introduction

Intrapulmonary percussive ventilation (IPV) is a ventilatory technique which is used to clear endobronchial secretions in patients. IPV uses a Phasitron, which delivers rapid, high-flow, minibursts of air mixed with oxygen to the patients. We investigated the safety of IPV on hemodynamic values and the effect of IPV on oxygen saturation and respiratory rate.

## Methods

From April until August 2011 we investigated 42 consecutive patients admitted to our eight-bed adult general ICU with respiratory failure. Variables such as heart rate, mean arterial pressure, respiratory rate, and oxygen saturation were measured and compared at three different time points: before starting IPV therapy, directly after and 15 minutes later. All patients received IPV using a Bird Intrapulmonary Percussionator Ventilator Model IPV-2C for a period of 20 minutes consisting of two cycles of 10 minutes. After the first 10 minutes of IPV therapy in combination with chest compressions the frequency rate was reduced in order to suction the mobilized secretions. This cycle was then repeated. Statistical analysis was done with SPSS version 17. Student's *t *test was used to compare values before therapy with directly after and after 15 minutes of therapy. *P *< 0.05 was considered significant.

## Results

Neither heart rate, mean arterial pressure nor respiratory rate showed any significant change after IPV. Oxygen saturation improved immediately after IPV and was also present after 15 minutes. See Table 1.

**Table 1:** Values before, after and 15 minutes after therapy

	Heart rate	Mean arterial pressure	Respiratory rate	Oxygen saturation
Before	84.5 ± 18.2	86.6 ± 19.0	24.7 ± 5.6	93.9 ± 3.0
After	86.0 ± 17.6	87.4 ± 21.0	24.1 ± 6.7	95.8 ± 2.8*
After 15 minutes	83.1 ± 16.7	85.4 ± 18.9	23.4 ± 6.0	95.5 ± 2.8*

*P < 0.01.

## Conclusion

We demonstrated that IPV is a safe therapy, and oxygen saturation improved after therapy with IPV.

